# Evolutionary diversity of the endemic genera of the vascular flora of Chile and its implications for conservation

**DOI:** 10.1371/journal.pone.0287957

**Published:** 2023-07-05

**Authors:** Pamela Ramírez-Verdugo, Alexis Tapia, Félix Forest, Rosa A. Scherson

**Affiliations:** 1 Herbario VALPL y Laboratorio de Botánica, Departamento de Ciencias y Geografía, Universidad de Playa Ancha, Valparaíso, Chile; 2 Jardín Botánico Nacional, Viña del Mar, Chile; 3 Laboratorio de Evolución y Sistemática, Departamento de Silvicultura y Conservación de la Naturaleza, Universidad de Chile, Santiago, Chile; 4 Departamento de Matemática, Universidad Técnica Federico Santa María, Valparaíso, Chile; 5 Jodrell Laboratory, Royal Botanic Gardens, Kew, Richmond, Surrey, United Kingdom; Southeastern Louisiana University, UNITED STATES

## Abstract

As a direct consequence of global change, both natural and human-induced, a high percentage of biodiversity is now under threat worldwide. This has urged conservation planners to formulate and/or improve existing strategies to preserve species and their ecosystems. In this context, the present study focuses on two strategies using phylogeny-based measures of biodiversity to account for the processes that led to the biodiversity patterns observed today. It will contribute additional information that can aid decision-making regarding the assignment of threat status for some species, thus strengthening measures currently in use and facilitate the allocation of often scarce conservation resources. The Evolutionarily Distinct (ED) index prioritises species that are on long branches of the tree of life with few descendants, and the Evolutionarily Distinct and Globally Endangered (EDGE) index integrates evolutionary history with IUCN Red List threat status of species. It has been used mostly in animal groups, but since the threats faced by many plants have not been evaluated, it has been more difficult to compile for plants worldwide. Here, we apply the EDGE metric to species of the endemic genera of Chile. However, more than 50% of the endemic flora of the country are still lacking official threat status. We thus used an alternative measure (Relative Evolutionary Distinctness–RED), based on a range-weighted phylogenetic tree, which uses geographic ranges to adjust branch lengths, and calculate ED. The RED index was shown to be a suitable measure, yielding similar results compared to EDGE, at least for this group of species. Given the urgency to halt biodiversity loss and the time it would take to evaluate all species, we propose that this index is used to set conservation priorities until we can calculate EDGE for these unique endemic species. This would allow guiding decision-making until we can gather more data to assess and assign conservation status to new species.

## Introduction

The Evolutionarily Distinct and Globally Endangered (EDGE) index has been instrumental for more than a decade in assigning conservation priorities for those taxa that are both evolutionarily distinct (i.e. found isolated on long branches on the tree of life with few close relatives) and under high levels of threat [1–4]. The "EDGE of Existence" program (https://www.edgeofexistence.org/) has promoted the protection of species that could in turn help protect evolutionary lineages with special, unique attributes, working toward the conservation of the future option values of biodiversity [5, 6]. The EDGE metric comprises two elements, ED and GE. The value of ED (Evolutionary Distinctness) for a given tip in the phylogenetic tree is obtained by summing up, for each branch from root to tip, the branch length divided by the number of species that descend from this branch [7]. ED assigns priority to those longer branches represented by a small number of taxa, under the assumption that those taxa are more evolutionarily unique. EDGE is calculated by integrating ED with the level of threat a particular taxon is facing, usually using the IUCN Red List categories as a surrogate for probability of extinction (the GE component). Therefore, EDGE assigns the highest priorities to threatened species that represent a significant amount of unique evolutionary history [8].

Here, we focused on calculating ED and EDGE for species of the endemic genera of Chile, a considerably threatened group of plants. Chile has been defined as a “biogeographic island” due to the geographic barriers that isolate its biota: the driest desert in the world to the north, the Andes mountain range to the east, the Pacific Ocean to the west and the Southern Ocean to the south [9]. This implies that biodiversity has developed in isolation with particular environmental conditions that have contributed to its high endemism. In plants, from a total of 4,655 native species, 40% are endemic to the country [10], presenting the highest number of endemic genera in the continent, and one of the highest in the world, with a total of 83–89 endemic genera [9, 11]. In addition, Chile is a very long country, with a variety of ecosystems or ecoregions [12, 13]. In the north we find the Atacama Desert, a very fragmented area ranging from an absolute desert in the central depression, to a desertic scrub in the coastal hills within a Mediterranean biome, and a high-altitude scrub (Altiplano) within a more Tropical biome. Central Chile (30° to 37° South) shows thorny and sclerophyllous forests, located from the coast to the Andes mountains up to 2,000 m and characterized by a Mediterranean-type weather with rainfall in the winter and a dry summer. The Mediterranean Andes show alpine vegetation up to 4,000 m. Temperate broad-leaved and deciduous rain forests are found below 35° South. Finally, the extreme south (Patagonia) shows an evergreen forest, steppe and grasslands with low average temperatures and strong winds. All ecoregions show different environmental conditions of altitude, temperature, rainfall and biodiversity [12, 14].

More than half the country has been defined as a biodiversity hotspot [15], characterized by its high level of endemism and threats due to human intervention, especially in the central area where most cities, industries and agricultural areas are located [16]. According to the latest National Biodiversity Report from the Ministry of the Environment of Chile [17], vascular plants are the most threatened group of organisms in the country. However, the conservation status of only 10% of them has been evaluated [18]. Conservation strategies are therefore necessary and urgent to preserve the unique flora found in the region.

Even though it is well-known that plants are one of the groups with the higher risk of extinction, mainly due to human activities, they remain one of the less well-known group of organisms in this respect. The reasons explaining this situation include the large numbers of species involved, complex taxonomic issues, the limited knowledge of geographic distribution in many cases, and the lack of phylogenetic studies [19]. The Global Strategy for Plant Conservation (GSPC) 2010–2020 (described in Lovett [20]) establishes in its second target that by 2020 we should have had “an assessment of the conservation status of all known plant species, as far as possible, to guide conservation action”. This goal is far from being complete. An evaluation from Lughadha et al. (2020) [21] estimated that only ca 10% of known plant species has been formally evaluated by IUCN, with 39% of the plant species in the world estimated to be threatened with extinction. In Chile, the situation is similar, with only 10% of the plants evaluated by the Ministry of the Environment. The high endemism of plant taxa in the country [9] will likely mean an even higher percentage of threatened species.

Measures that include evolutionary contribution such as ED and EDGE have been very useful and increasing in the last decade. Even though the utility of the EDGE index has been well established [4], it has been an especially difficult task in plants for the above-mentioned reasons. Globally for plants, only cycads [22], gymnosperms [2], and a small clade of yams [23] have been evaluated to date using the EDGE approach. To our knowledge, no EDGE evaluation has been published using regional red list assessments. In 2010, the Ministry of the Environment in Chile established that the local red lists should follow the exact same criteria as the IUCN Red List and establish the same threat categories. This means that this local list is comparable to the IUCN Red List. Evaluations done with these types of red lists are thus also comparable with others in different parts of the world.

The Global Biodiversity Outlook released in 2020 by the Convention of Biological Diversity (CBD) reported how most of the 2011–2020 Aichi targets have not been fully met, leading to a new set of goals (post-2020 framework) recently convened upon during the Kunming–Montreal Global Biodiversity Framework. The CBD states that we need a “transformative change leading to a new relationship with biodiversity”. Halting biodiversity loss is one of the most pressing goals in this respect. However, in order to prioritize those species that need the most attention, we need data on the conservation status of all species to be able to incorporate them in prioritization programs such as EDGE. The process of obtaining the necessary data needed to achieve this can be arduous, especially for countries in which funding for this type of research is scarce [24]. Limited data, coupled with the urgency to halt biodiversity loss, calls for methods that can surrogate EDGE calculations until data on conservation status for all species becomes available. These temporary surrogates could help highlighting those taxa that need the most urgent conservation attention and could thus speed up conservation assessments. Here, we propose an approach that provides temporary EDGE-like values when threat data are not available, through a new index, Relative Evolutionary Distinctness (RED). A species’ geographic distribution is an important criterion to establish its conservation status and there is often enough distribution data for a given species even if it has not been formally evaluated. In fact, geographic range has been described as one of the most important correlates to extinction risks [25, 26]. A range-weighted tree is a phylogenetic tree in which branch-lengths are scaled proportionally to the geographic distribution of the terminals, and is an approach commonly used to calculate phylogenetic endemism and other phylogeny-based measures [27, 28]. We report here the utility of this approach as a source of EDGE-like values for a group of endemic plant species from Chile with little information on their conservation status.

## Materials and methods

### Taxon sampling

We analyzed 197 species, representing 85% of the total number of species found in the endemic vascular plant genera of Chile [11]. This particular set of species have well-studied distributions, and even though many of them do not have threat status assessments, the percentage of evaluated species is higher than the rest of the native species. In addition, because for endemic species, local assessments are equivalent to global ones, this particular group of species appear as an adequate sample to perform these analyses. We obtained sequences for 147 species from GenBank (National Center for Biotechnology Information), and direct sequencing from plant material gathered from indexed herbaria and direct field collections. Species that could not be sequenced due to lack of material or problems with the PCR reaction were randomly included in the phylogenetic tree (see below for details). DNA extraction for field-collected material was performed using the DNeasy® Plant Mini Kit (QIAGEN Inc) according to the manufacturer’s recommendations. We used the *rbcL* and *trnL-F* plastid regions, amplifying each one by PCR, according to [29]. Primers used were *rbcL1F* and *rbcL1352R* for *rbcL* and trnL-F_F and trnL_R for the *trnL-trnF* spacer [30]. These markers were chosen because of the availability of sequences in GenBank, and the fact that alignments are more accurate with these markers than for example ITS when a broad range of plant taxa are sampled. The final PCR product was purified and sequenced using Applied Biosystems ABI3700 and ABI3730 XL at Macrogen Inc. in South Korea. Chromatographs were analyzed, and contigs were assembled using Sequencher version 5.4 (Gene Codes Corporation).

Alignments for each marker were compiled using the software MAFFT version 7.0 [31] and AliView version 3.0 [32], with the L-INS-I algorithm, and were combined in a single matrix using Mesquite 3.03 [33]. The outgroup taxon used was *Thyrsopteris elegans* (Monilophyte), whose sequences were obtained from GenBank (accession numbers for *rbcL* HG422549 and for *trnL-F* HG422548). A constraint phylogenetic tree at the family level was first reconstructed based on the APGIV schematic tree [34]. Phylogenetic reconstruction was conducted using maximum likelihood as implemented in RAxML-Light [35] on the CIPRES Science Gateway platform (https://www.phylo.org/). The GTR+I+G model of evolution was used for the phylogenetic analysis. Statistical support for nodes was obtained by bootstrap analysis with 1,000 replicates; clades with bootstrap values above 90% were considered well-supported. The tree was made ultrametric using the function *chronos* from the R package ape [36] and the root was calibrated by assigning it a value of 100 after pruning the outgroup taxon *Thyrsopteris elegans*.

The geographic data matrix was built using geographic coordinates for all species, obtained from Scherson et al (2017) [37] complemented with field data, and were projected on a 0.5 by 0.5-degree grid. The database is available as Supporting Information and deposited in Dryad (https://datadryad.org/). For further details regarding this database, see [37].

### Addition of missing species

As DNA sequence data was not available for all species, we used two approaches to incorporate the missing species in our backbone phylogenetic tree. First, for missing species with only one representative in the study (or from monotypic genera) and for which the genus was not represented in the backbone tree, we added them manually based on information obtained from the literature, assigning them to the mid-point of a given branch. These included *Ivania juncalensis* (sister to the genus *Aimara*; [10, 38]), *Kieslingia chilensis* (sister to the genus *Guynesomia*; [39], *Yunquea tenzii* (sister to the genus *Centaurodendron*; [40]), *Gymnachne jaffuelii* and *G*. *koelerioides* (in the *Megalachne* clade; [40]), *Nesocaryum stylosum* (in the *Cuminia* clade; [10]), *Selkirkia berteroi* (in the *Cuminia* clade [41]), and *Lycapsus tenuifolius* (in the same clade as *Leptocarpha* and *Podanthus*; [10]). The rest of the missing species were from genera with other representatives already in the phylogenetic tree. In these cases, we used the function *add*.*species*.*to*.*genus* from the R package phytools (R development Core Team 2018 [42, 43]) with the option “random”, which randomly adds the missing species to their respective genera (as in [2]). This procedure was replicated 100 times to account for the phylogenetic uncertainty linked to the inclusion of missing species; the resulting set of 100 phylogenetic trees was used in subsequent analyses.

### Calculation of evolutionary indices

Evolutionary Distinctness (ED) and Evolutionarily Distinct and Globally Endangered (EDGE) indices were calculated from the 100 phylogenetic trees (see above) using the Tuatara package implemented in Mesquite version 3.61 [33], according to Isaac et al. [1]. Median values were compiled from the scores obtained from the 100 trees. For the EDGE measurements, for all plants that have been evaluated, IUCN conservation status was converted to probabilities of extinction using the indices of extinction probabilities provided by IUCN for 50, 100 and 500 years, as suggested by Mooers et al. [44], and the probabilities proposed by Isaac et al. [1] for 100 years. Conservation status were sourced from the list compiled by the Chilean Ministry of the Environment (https://clasificacionespecies.mma.gob.cl), which follows the same criteria established by IUCN.

**Fig 1:**
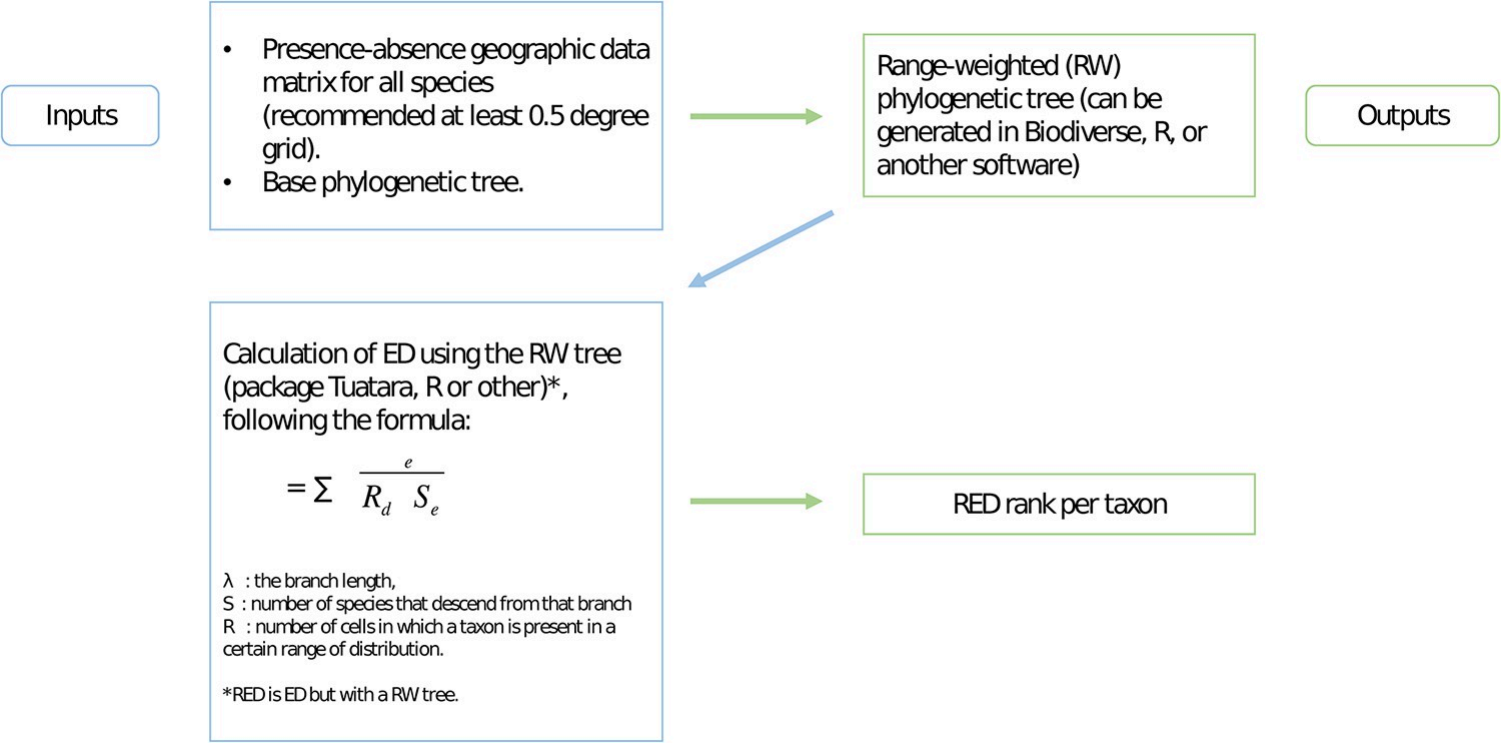
Steps to calculate the RED index.

The Relative Evolutionary Distinctness (RED) index proposed here was calculated for evaluated and non-evaluated species. For this, we used a presence-absence geographic data matrix for all species, using a 0.5-degree geographic grid covering all of Chile. This, together with the phylogenetic tree, were uploaded into Biodiverse 3.0 [45] to obtain a range-weighted phylogenetic tree in which the length of each branch is divided by the number of grid cells in which the species that descend from it are present [27]. In this range-weighted tree, the branches that subtend taxa with narrow distributions remain equal or very similar in length, because the branch length is divided by a low number, while branches that subtend taxa that are widely distributed are divided by a large number (number of grid cells were a taxon or a group of taxa is present) and are thus proportionately shorter. This tree was then used as input for ED calculations in Tuatara. So basically, RED is ED calculated on a range-weighted tree. As stated in the methodology for ED [27], taxa with the highest ED values are those in long branches with few relatives, whereas taxa that are in short branches and have more taxa that share those branches, will have a lower ED value (Fig 1). All analyses were done for 100 trees and the median value for each index was considered for all statistical correlations. RED values do not have a meaning in themselves, what is relevant is their position in a ranking, which is the same method used for ED and EDGE.

### Statistical validation of the RED index

In order to validate the RED index, a statistical comparison between EDGE and RED for evaluated species was carried out. Median values for the EDGE index were compared with the median values for RED for all evaluated species. We used two statistical indicators, the Kendall (Kendall W) [46] concordance coefficient and a Spearman [46] simple range correlation. The Kendall W coefficient was used as a measure of agreement between both indices. This coefficient is useful because it compares ranges instead of raw values.

In addition, a Spearman simple range correlation was used. This test allows measuring the correlation or association of two variables and it can be applied for range correlations.

## Results

**Fig 2:**
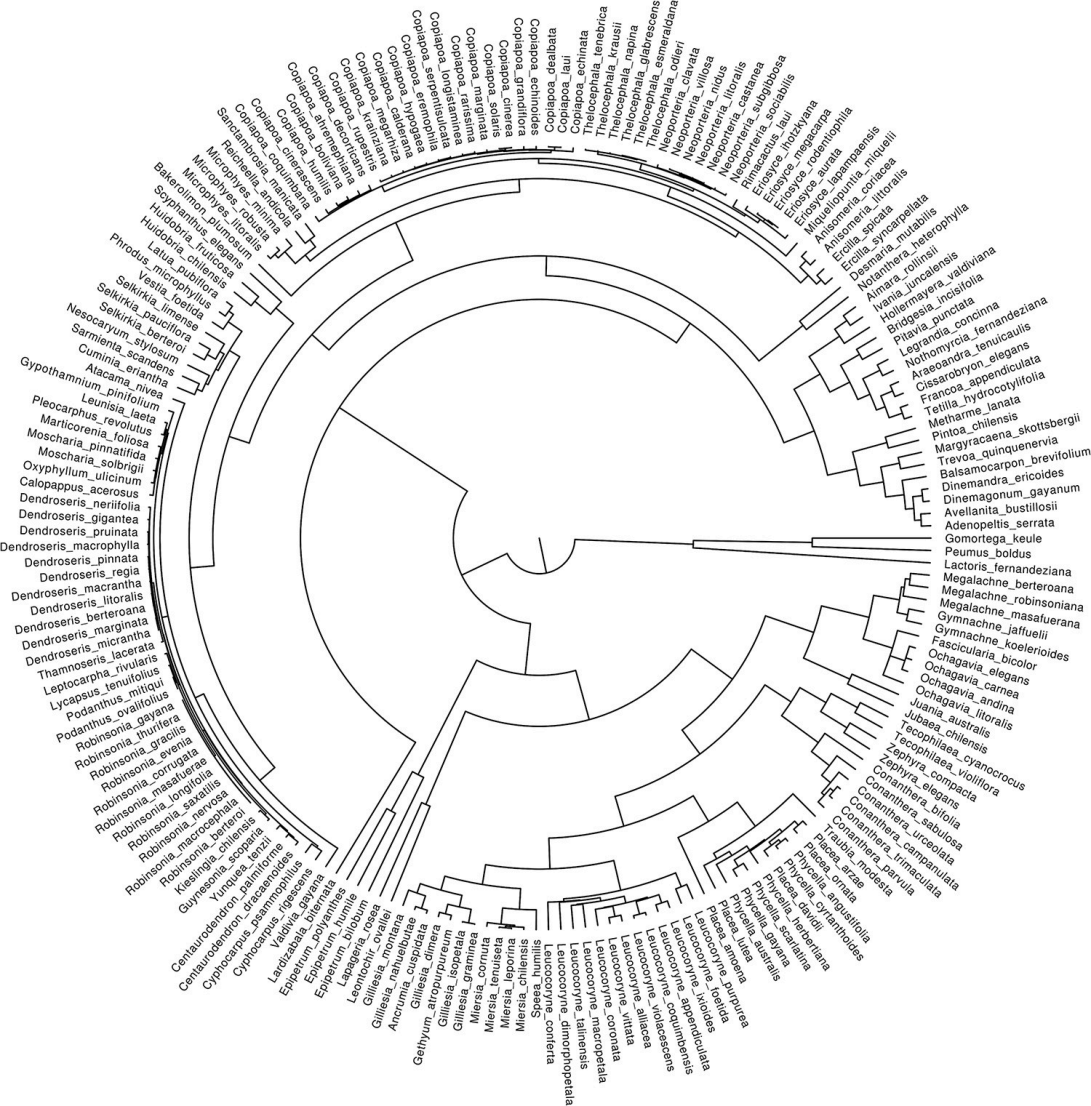
One of the 100 maximum likelihood phylograms randomly selected, obtained from *rbcL* and *trnL-F* (and made ultrametric) of the species belonging to the endemic genera of vascular flora of Chile to which missing species were added (see methods for details).

We obtained sequences for 147 species, representing 92% of the genera of vascular plants that are endemic to Chile. From those genera, we managed to obtain sequences for 63% of the species. After adding species with no sequences (see methods), our matrix covered 100% of the endemic genera, and 85% of their species. The final tree contained 197 taxa (Fig 2). Alignments and phylogenies obtained are available in Dryad (https://datadryad.org/).

From the total number of species in this study, only 95 species (41%) are classified in some of the IUCN Red List categories. A large proportion of the species classified are listed as threatened (84 species, 88%): Critically Endangered, 7 species, 7%; Endangered, 54 species, 57%; Vulnerable, 23 species, 24%; Least Concern, 4 species, 4%; and Near Threatened, 7 species, 7%. Most of the island species have been evaluated for conservation status (83%), however, only 32% of the continental species have conservation assessments assigned to them; 130 species from this area have not yet been evaluated (NE).

Details of the conservation status of the sampled species are provided in Table 1.

**Table 1:** General statistics of the conservation status of the sampled species analyzed in this study.

Threat category of the studied species.	Quantity	Percent
Critically endangered (CR)	7	7%
Endangered (EN)	54	57%
Vulnerable (VU)	23	24%
Near Threatened (NT)	7	7%
Least Concern (LC)	4	4%
**TOTAL**	**95**	**100%**

### Calculated indices

We produced conservation priority lists for the species belonging to the endemic genera of the vascular flora of Chile that account for their evolutionary history. The first index calculated was ED. Table 2 shows the top 20 scores (30 species).

**Table 2:** List of the top 20 ED scores obtained with the median values of 100 trees. Conservation status and geographic distribution are provided.

Species (Family)	ED Rank	Conserv. status (when known)	Geographic distribution
*Lactoris fernandeziana* (Lactoridaceae)	1	EN	JFA
*Gomortega keule* (Gomortegaceae)	2	EN	CC
*Peumus boldus* (Monimiaceae)	2		CC
*Epipetrum bilobum* (Dioscoreaceae)	3		CC
*Epipetrum humile* (Dioscoreaceae)	4		CC
*Lapageria rosea* (Philesiaceae)	5		CC
*Leontochir ovallei* (Alstroemeriaceae)	5	EN	CC
*Lardizabala biternata* (Lardizabalaceae)	6		CC
*Epipetrum polyanthes* (Dioscoreaceae)	7		CC
*Juania australis* (Arecaceae)	8	EN	JFA
*Jubaea chilensis* (Arecaceae)	8	VU	CC
*Desmaria mutabilis* (Loranthaceae)	9		CC
*Notanthera heterophylla* (Loranthaceae)	9		CC
*Placea amoena* (Amaryllidaceae)	10	EN	CC
*Tecophilaea cyanocrocus* (Tecophilaeaceae)	11	EN	CC
*Tecophilaea violiflora* (Tecophilaeaceae)	11		CC
*Conanthera bifolia* (Tecophilaeaceae)	12		CC
*Zephyra compacta* (Tecophilaeaceae)	13		CC
*Zephyra elegans* (Tecophilaeaceae)	13		CC
*Metharme lanata* (Zygophyllaceae)	14	EN	CC
*Pintoa chilensis* (Zygophyllaceae)	14	EN	CC
*Scyphanthus elegans* (Loasaceae)	15		CC
*Hollermayera valdiviana* (Brassicaceae)	16		CC
*Bakerolimon plumosum* (Plumbaginaceae)	17		CC
*Trevoa quinquenervia* (Rhamnaceae)	18		CC
*Bridgesia incisifolia* (Sapindaceae)	19		CC
*Pitavia punctata* (Rutaceae)	19	EN	CC
*Huidobria chilensis* (Loasaceae)	20		CC
*Huidobria fruticosa* (Loasaceae)	20		CC

JFA = Juan Fernandez Archipelago; CC = Continental Chile. Taxa with identical ED scores show equal rank status (see for example *Gomortega keule* and *Peumus boldus* that occupy the 2nd rank)

EDGE ranks are presented in Table 3 for different extinction probability scenarios and compared with the calculated RED scores for all evaluated species. With each of the EDGE indices, RED shares 65% of the species in the top 20 presented.

**Table 3:** Top 20 ranks for RED and EDGE indices, according to the median values obtained for 100 trees, conservation status and geographic distribution.

*Species* (Conserv. Status) Family	Rank RED	EDGE IUCN50	Rank EDGE IUCN50	EDGE IUCN100	Rank EDGE IUCN100	EDGE IUCN500	Rank EDGE IUCN500	EDGE Isaac	Rank EDGE Isaac
***Lactoris fernandeziana*** (EN) Lactoridaceae	1	***Lactoris fernandeziana*** (EN) Lactoridaceae	1	***Lactoris fernandeziana*** (EN) Lactoridaceae	1	***Lactoris fernandeziana*** (EN) Lactoridaceae	1	***Lactoris fernandeziana*** (EN) Lactoridaceae	1
***Juania australis*** (EN) Arecaceae	2	***Gomortega keule*** (EN) Gomortegaceae	2	***Gomortega keule*** (EN) Gomortegaceae	2	***Gomortega keule*** (EN) Gomortegaceae	2	***Gomortega keule*** (EN) Gomortegaceae	2
***Tecophilaea cyanocrocus*** (EN) Tecophilaeaceae	3	***Leontochir ovallei*** (EN) Alstroemeriaceae	3	***Leontochir ovallei*** (EN) Alstroemeriaceae	3	***Leontochir ovallei*** (EN) Alstroemeriaceae	3	***Leontochir ovallei*** (EN) Alstroemeriaceae	3
***Placea amoena*** (EN) Amaryllidaceae	4	***Juania australis*** (EN) Arecaceae	4	***Megalachne masafuerana*** (CR) Poaceae	4	***Juania australis*** (EN) Arecaceae	4	***Juania australis*** (EN) Arecaceae	4
***Leontochir ovallei*** (EN) Alstroemeriaceae	5	***Placea amoena*** (EN) Amaryllidaceae	5	***Juania australis*** (EN) Arecaceae	5	***Placea amoena*** (EN) Amaryllidaceae	5	***Megalachne masafuerana*** (CR) Poaceae	5
***Placea lutea*** (EN) Amaryllidaceae	6	***Tecophilaea cyanocrocus*** (EN) Tecophilaeaceae	6	***Placea amoena*** (EN) Amaryllidaceae	6	***Tecophilaea cyanocrocus*** (EN) Tecophilaeaceae	6	***Placea amoena*** (EN) Amaryllidaceae	6
***Megalachne masafuerana*** (CR) Poaceae	7	***Megalachne masafuerana*** (CR) Poaceae	7	***Cuminia eriantha*** (CR) Lamiaceae	7	***Metharme lanata*** (EN) Zygophyllaceae	7	***Tecophilaea cyanocrocus*** (EN) Tecophilaeaceae	7
*Megalachne berteroana* (VU) Poaceae	8	***Metharme lanata*** (EN) Zygophyllaceae	8	***Tecophilaea cyanocrocus*** (EN) Tecophilaeaceae	8	***Pintoa chilensis*** (EN) Zygophyllaceae	7	***Cuminia eriantha*** (CR) Lamiaceae	8
***Cuminia eriantha*** (CR) Lamiaceae	9	***Pintoa chilensis*** (EN) Zygophyllaceae	8	***Sanctambrosia manicata*** (CR) Caryophyllaceae	9	*Pitavia punctata* (EN) *Rutaceae*	8	***Metharme lanata*** (EN) Zygophyllaceae	9
***Metharme lanata*** (EN) Zygophyllaceae	10	*Pitavia punctata* (EN) *Rutaceae*	9	***Metharme lanata*** (EN) Zygophyllaceae	10	***Megalachne masafuerana*** (CR) Poaceae	9	***Pintoa chilensis*** (EN) Zygophyllaceae	9
***Sanctambrosia manicata*** (CR) Caryophyllaceae	11	***Cuminia eriantha*** (CR) Lamiaceae	10	***Pintoa chilensis*** (EN) Zygophyllaceae	10	***Placea lutea*** (EN) Amaryllidaceae	10	***Sanctambrosia manicata*** (CR) Caryophyllaceae	10
***Gomortega keule*** (EN) Gomortegaceae	12	***Sanctambrosia manicata*** (CR) Caryophyllaceae	11	*Pitavia punctata* (EN) *Rutaceae*	11	*Conanthera urceolata* (EN) Tecophilaeaceae	11	*Pitavia punctata* (EN) *Rutaceae*	11
*Nothomyrcia fernandeziana* (VU) Myrtaceae	13	***Placea lutea*** (EN) Amaryllidaceae	12	***Nesocaryum stylosum*** (CR) Boraginaceae	12	*Jubaea chilensis* (VU) Arecaceae	12	*Jubaea chilensis* (VU) Arecaceae	12
***Nesocaryum stylosum*** (CR) Boraginaceae	14	*Conanthera urceolata* (EN) Tecophilaeaceae	13	***Placea lutea*** (EN) Amaryllidaceae	13	*Legrandia concinna* (EN) Myrtaceae	13	***Placea lutea*** (EN) Amaryllidaceae	13
*Leucocoryne foetida* (VU) Alliaceae	15	*Legrandia concinna* (EN) Myrtaceae	14	*Conanthera urceolata* (EN) Tecophilaeaceae	14	*Avellanita bustillosii* (EN) Euphorbiaceae	14	***Nesocaryum stylosum*** (CR) Boraginaceae	14
*Rimacactus laui* (EN) Cactaceae	16	*Avellanita bustillosii* (EN) Euphorbiaceae	15	*Legrandia concinna* (EN) Myrtaceae	15	***Cuminia eriantha*** (CR) Lamiaceae	15	*Conanthera urceolata* (EN) Tecophilaeaceae	15
*Valdivia gayana* (VU) Escalloniaceae	17	*Gethyum atropurpureum* (EN) Alliaceae	16	*Avellanita bustillosii* (EN) Euphorbiaceae	16	*Gethyum atropurpureum* (EN) Alliaceae	16	*Legrandia concinna* (EN) Myrtaceae	16
*Ochagavia elegans* (VU) Bromeliaceae	18	***Nesocaryum stylosum*** (CR) Boraginaceae	17	*Gethyum atropurpureum* (EN) Alliaceae	17	***Sanctambrosia manicata*** (CR) Caryophyllaceae	17	*Avellanita bustillosii* (EN) Euphorbiaceae	17
***Pintoa chilensis*** (EN) Zygophyllaceae	19	*Miersia cornuta* (EN) Alliaceae	18	*Miersia cornuta* (EN) Alliaceae	18	*Miersia cornuta* (EN) Alliaceae	18	*Gethyum atropurpureum* (EN) Alliaceae	18
*Yunquea tenzii* (EN) Asteraceae	20	*Microphyes robusta* (EN) Caryophyllacee	19	*Microphyes robusta* (EN) Caryophyllacee	19	***Nesocaryum stylosum*** (CR) Boraginaceae	19	*Miersia cornuta* (EN) Alliaceae	19
		*Selkirkia berteroi* (EN) Boraginaceae	20	*Neoporteria sociabilis* (CR) Cactaceae	20	*Microphyes robusta* (EN) Caryophyllacee	20	***Megalachne berteroana*** (VU) Poaceae	20

For EDGE, probabilities of extinction were assigned according to reduction in population abundance estimated in a maximum of 50 years (IUCN 50), 100 years (IUCN 100), 500 years (IUCN 500) and probabilities of extinction assigned by Isaac et al. [1]. The species present in both lists (RED and EDGE) are marked in bold. JFA = Juan Fernandez ***Archipelago***; ID = Islas Desventuradas; CC = Continental Chile.

**Fig 3:**
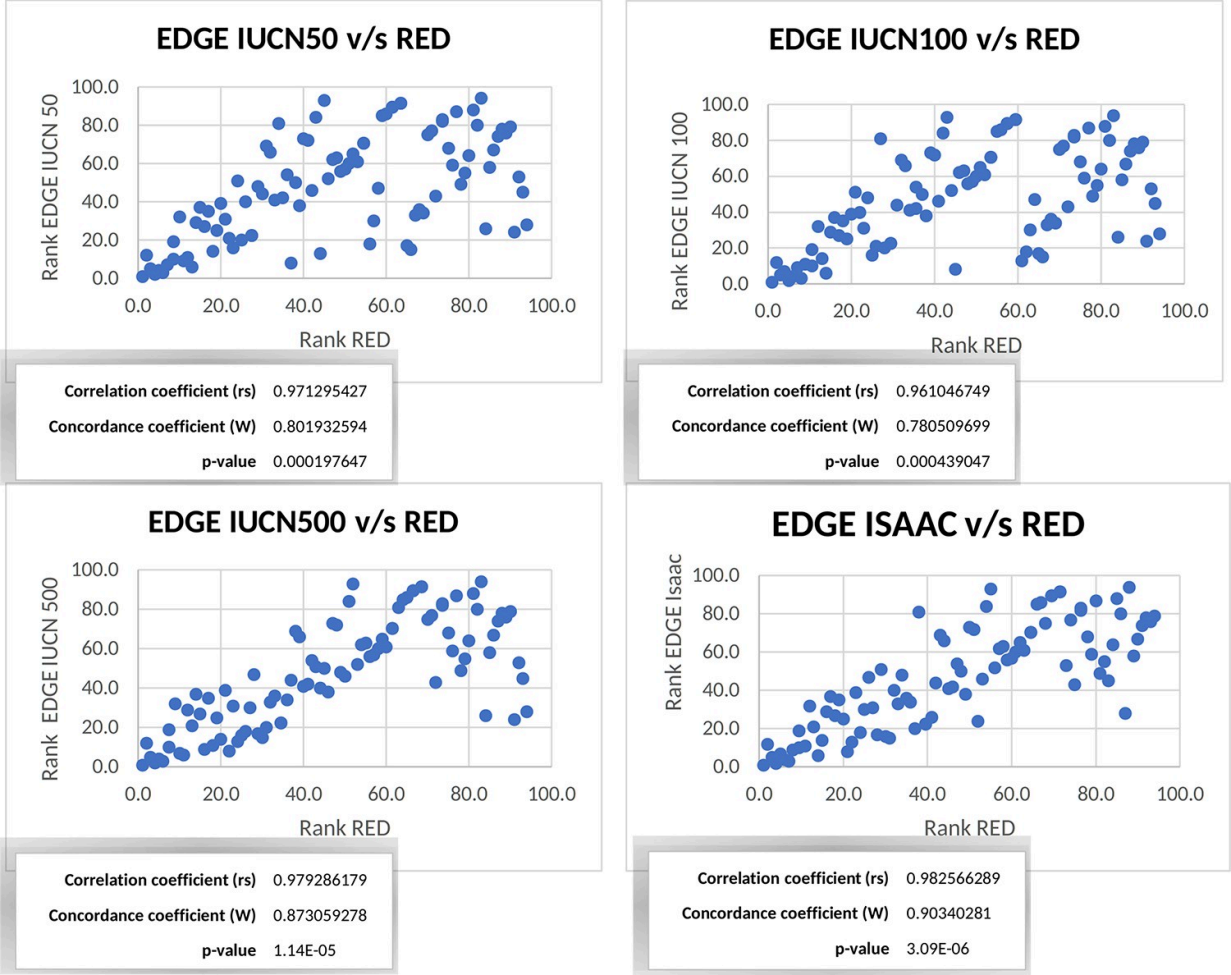
Correlation graph between EDGE and RED ranks, calculated for species with conservation category and their weighting assigned by the IUCN with an estimated of reduction of population in a maximum of 50 years (IUCN50), 100 years (IUCN100), 500 years (IUCN500) and probabilities of extinction assigned by Isaac et al. [1].

Both the Kendall (W) concordance coefficient and the Spearman correlation coefficient (rs) yielded high correlation between the EDGE and RED indices (p<0.001 for all analyses). The Kendall concordance coefficient (W), Spearman correlation coefficient (rs) and associated p-values are presented in Fig 3 with their respective correlation graph, for each set of extinction probabilities.

Given the high correlation shown for the EDGE and RED indices, we calculated RED for all species regardless of whether they were evaluated. The top 20 RED scores are presented in Table 4.

**Table 4:** Top 20 list of RED for species with and without conservation status, showing the RED rank, median RED value for 100 trees, conservation status when available and geographic distribution.

Species	Rank RED	Conserv. status (when known)	Geographic distribution
*Lactoris fernandeziana*	1	EN	AJF
Lactoridaceae			
*Epipetrum polyanthes*	2		Chile Cont.
Dioscoreaceae			
*Epipetrum bilobum*	3		Chile Cont.
Dioscoreaceae			
*Juania australis*	4	EN	AJF
Arecaceae			
*Tecophilaea cyanocrocus*	5	EN	Chile Cont.
Tecophilaeaceae			
*Zephyra compacta*	6		Chile Cont.
Tecophilaeaceae			
*Placea amoena*	7	EN	Chile Cont.
Amaryllidaceae			
*Epipetrum humile*	8		Chile Cont.
Dioscoreaceae			
*Leontochir ovallei*	9	EN	Chile Cont.
Alstroemeriaceae			
*Placea lutea*	10	EN	Chile Cont.
Amaryllidaceae			
*Conanthera sabulosa*	11		Chile Cont.
Tecophilaeaceae			
*Megalachne masafuerana*	12	CR	AJF
Poaceae			
*Megalachne berteroana*	13	VU	AJF
Poaceae			
*Cuminia eriantha*	14	CR	AJF
Lamiaceae			
*Ivania juncalensis*	15		AJF
Brassicaceae			
*Megalachne robinsoniana*	16		AJF
Poaceae			
*Metharme lanata*	17	EN	Chile Cont.
Zygophyllaceae			
*Hollermayera valdiviana*	18		Chile Cont.
Brassicaceae			
*Sanctambrosia manicata*	19	CR	ID
Caryophyllaceae			
*Gymnachne jaffuelii*	20		Chile Cont.
Asteraceae			

JFA = Juan Fernandez Archipelago; ID = Islas Desventuradas; CC = Continental Chile.

## Discussion

Our goal in this study was to obtain EDGE and RED scores for plant species belonging to endemic genera of vascular plants of Chile. The rationale for using only the endemic genera is two-fold. On one hand, local red list assessments are evidently also global in scope because these species are endemic to Chile and found nowhere else. This is the case for a large proportion of plant species worldwide [47]. However, because of the lack of evaluation for so many species due to limited phylogenetic and conservation status data, EDGE lists for plants will remain incomplete for the foreseeable future except if particular efforts are made to rectify this situation using various approaches to account for missing information (e.g. machine learning) [48]). The urgency to conserve those species is far more pressing than our ability to produce evaluations, which justifies the need for surrogates until all species are evaluated as is the case for other taxa such as mammals [1, 49], reptiles [50], amphibians [48, 51] or birds [52, 53] (see http://edgeofexistence.org/ [8]).

We showed that our RED index can be used as an EDGE-like measure. In the validation experiment, in which we compared RED with EDGE only for evaluated species, from the 20 top species ranked for RED, 13 (65%) are also in all the EDGE lists regardless of the extinction probabilities used. However, there are six species that are in the RED top 20 list (Table 3) but in none of the EDGE top 20 lists. These species are in different conservation categories, so that is probably not a factor influencing their placement. Their common characteristic is their very restricted distribution in Chile, a factor that likely influences the relative weight of the branches of the tree when calculating RED, probably overestimating their ranking.

Two species present in all lists show a curious pattern of ranking. *Pintoa chilensis* (Zygophyllaceae) tends to get pushed to the bottom of the ranking for RED as opposed to EDGE where it is in the top part of the ranking for all extinction probability transformations used. *Placea lutea* (Amaryllidaceae) shows the opposite behaviour; while it is in the top section of the RED ranking, it occupies lower rankings in the EDGE lists, regardless of the extinction probability transformations used. This is likely due to the distribution range of these species. *Pintoa chilensis* is more widely distributed than *Placea lutea*, which probably explains the difference in placements in the RED versus EDGE priority ranking.

Statistically, the validation is very significant, with high correlation coefficients for all comparisons. Because both statistical indices used are based on comparisons of ranks, we can assume that the placement of species in rank intervals is very similar between indices. This implies that it is possible to perform a prioritization using RED for those species that have not yet been assigned a conservation status.

For this study, we used a tree containing all species of the endemic genera of Chile. When considering species from a broad range of vascular plants, the ideal tree to be used is the complete tree of all vascular plant species. However, because the RED index uses distribution data, we would not have accurate information for all species of vascular plants. Since the purpose of this study was to offer a comparison between EDGE and RED, we focused on using the most information available and on calculating both indices on comparable sets of data, i.e. on the same tree. The importance of this study is the delivery of an index that can be used until threat status becomes available for all species. The EDGE2 protocol, a recently published update of the original EDGE approach, allows the inclusion of non-evaluated and data deficient species and account for the uncertainty in the quantification of extinction risk by sampling a distribution of probabilities of extinction [54]. While this approach allows the compilation of EDGE scores for groups with a large proportion of species without a Red List assessment, the level of uncertainty introduced by many unassessed species remains relatively important. Thus, RED offers an interim mean to address this situation. As a precautionary note, we noticed that the correlation between RED and EDGE is very strong in the top 40–50 places of the ranking but becomes weaker in lower rankings. We thus recommend considering RED in its higher range of values, to highlight those species that need to be considered as priorities.

Given that RED uses a range-weighted tree, it allows the prioritization of species with restricted distribution, one of the important criteria when defining conservation categories. However, more information is always desirable. IUCN conservation categories often also consider information on populations such as genetic diversity, reproductive strategies and success, fitness, and very importantly, threats. Therefore, EDGE should always be preferred when IUCN categories are available, because the GE component is based on far more information than only distribution range.

Our RED ranking made with both evaluated and non-evaluated species contains non-evaluated species in high priorities. The high proportion of monocots among these species is worth noting. Examples of these are three species of the family Dioscoreaceae (*Epipetrum polyanthes*, *Epipetrum bilobum*, *Epipetrum humile*), two species of Tecophilaeaceae (*Zephyra compacta*, *Conanthera sabulosa*), and *Megalachne robinsoniana* (Poaceae). Other non-evaluated species include *Ivania juncalensis* and *Hollermayera valdiviana* (Brassicaceae). Some of these species have already been indicated as candidates for the evaluation process. For example, *E*. *bilobum* is endemic to an area of the Atacama Desert and according to Finger and Tellier (2010) [55], should be considered Vulnerable due to its narrow distribution range and the anthropogenic pressures on the area where it grows.

Even though RED can provide valuable information about species that have not been evaluated, it can also be useful to re-consider species that have been assessed, but whose evolutionary contribution has not been accounted for. Some of the evaluated species with low levels of threat (LC or NT) appear in our RED ranking in the top half. ED adds the evolutionary component, but there are differences in the rank estimates for both indices, due to the fact that RED also considers distribution ranges.

The EDGE index only considers terminal taxa in isolation and can thus overestimate the amount of unique evolutionary history under threat by failing to account for the status of close relatives in the phylogenetic tree (i.e., the amount of evolutionary history at risk through the loss of a threatened species is less if its close relatives are not imperilled [56]). Approaches such as the heightened EDGE (HEDGE) metric [57] and the recently published EDGE2 protocol [54] have been developed that account for the extinction risk of close relatives [58]. However, for this study, we aimed at developing a simple and provisional EDGE-like index that could serve as a guide to identify species in urgent need of attention.

The need for preserving biodiversity is evident and urgent, due to the speed at which human activities are damaging natural environments. However, limited resources force decision-makers to set priorities in terms of what to preserve based on the resources available [59]. Mace et al. (2003) [60] have indicated that many of the problems facing conservation, regardless of the focus, stem from lack of knowledge. Clearly, if precise distribution information was available for every species on the planet and if their threats could be periodically evaluated, biodiversity conservation would be a much simpler task. However, this is far from being the case. If we decided that we would only carry out conservation projects that have 100% of available information, we would protect very few species [6]. Given this situation, we highlight the importance of using available data to protect biodiversity in an era of extinction.

There are already some proposals that could accelerate the assessments of the conservation status of species, based on strategies that work with less data, or using data that we can find more easily on platforms such as the Global Biodiversity Information Facility (GBIF; www.gbif.org) or POWO (http://www.plantsoftheworldonline.org), using open-source information. Examples of these are the Rapid Least Concern strategy [61], the models proposed by Darrah et al. (2017) [62], or the iucn_sim approach [46], with open-source command lines, which simulate future extinctions using information on the evaluation history and estimated state transition rates, obtained from IUCN status of the last decades. However, none of these new approaches include information on evolutionary history. This information could be easily available given the speed at which DNA sequences and phylogenetic trees are being generated. In addition, most of these studies now also demand the geographic data to be publicly available through online platforms such as GBIF. These data could improve species conservation, integrating existing evaluations with evolutionary information, such as has been done with EDGE or the index RED presented here, which are able to protect not only species, but also their genetic heritage and with that, future option values [63].

Indices such as the one proposed here could be very useful to obtain global perspectives on threatened plant taxa, considering the extensive threats to which ecosystems are subjected to. In Chile, 3,5% to 4,5% of native forest is lost every year [17, 64], especially in the central area of the country. In addition, evaluated species only cover a small percentage of the flora of the country, a common situation for many areas in the world [65, 66]. Finally, evolutionary indices are not static, they can be adjusted as new information becomes available, such as conservation categories and geographic distribution.

## Supporting information

S1 Table Endemic genera species considered valid in this study, species analyzed for EDGE and RED parameters used to test sensitivity of ranks-to-extinction probability transformations and GenBank accession numbers. (XLSX)

S2 Table ED, RED and EDGE scores for species with and without conservation category. (XLSX)

S3 Table Summary of correlation and concordant coefficients of EDGE versus RED for four different scenarios of extinction probabilities. (XLSX)

S4 Table Correlation graphs between EDGE and RED, considering the different probability of extinction scenarios described in the methods. (XLSX)

S1 Data Phylogenetic tree in Newick format used for the calculations. (PHY)
